# Rebound pain following peripheral nerve block in extremity fracture surgery: pathophysiological mechanisms based on the “Triple-Hit” model and multimodal preventive strategies

**DOI:** 10.3389/fmed.2026.1817032

**Published:** 2026-05-05

**Authors:** Feng Zhou, XiaoLi Zhu, Jinsen Liu, Lin He, Longsheng Zhao, Jianhong Xu

**Affiliations:** Department of Anaesthesiology, The Fourth Affiliated Hospital of School of Medicine, International School of Medicine, International Institutes of Medicine, Zhejiang University, Yiwu, China

**Keywords:** rebound pain, peripheral nerve block, extremity fracture, multimodal analgesia, Triple-Hit Model

## Abstract

Extremity fracture surgery is the standard surgical intervention for traumatic musculoskeletal injuries. The maturation of ultrasound-guided visualization techniques has facilitated the widespread application of peripheral nerve block (PNB) in perioperative anesthesia and analgesia. However, rebound pain (RP)—defined as the phenomenon wherein previously suppressed nociceptive signals exhibit abrupt intensification exceeding baseline levels following the termination of regional analgesic effects—has emerged as a significant clinical challenge. RP interferes with early postoperative functional mobilization, compromises patient satisfaction, and increases healthcare resource utilization. The pathogenesis of RP involves a multifactorial pathophysiological process: hyperexcitability of nerve fibers following block resolution; compensatory neurophysiological responses subsequent to local anesthetic pharmacodynamic decline; iatrogenic neural effects attributable to needle instrumentation or surgical manipulation; localized inflammatory cascade activation triggered by tissue trauma; and interindividual variability in genetic susceptibility and psychological resilience. The cornerstone of RP prevention lies in the multimodal analgesic concept, integrating pharmacological agents and techniques with divergent mechanisms of action. This review proposes a conceptual “Triple-Hit Model of RP,” hypothesizing that RP arises from the dynamic interplay of three interrelated risk elements: the first element is the magnitude of initial peripheral nociceptive input, predominantly determined by surgical trauma severity; the second element is individual central nervous system modulation capacity and pain tolerance thresholds, influenced by chronological age, anxiety states, and related factors; the third element is the withdrawal pattern of regional analgesic protection, encompassing block duration and offset velocity. The synergistic convergence of these three hits substantially amplifies the risk of severe RP manifestation.

## 1 Introduction

Extremity fractures represent common traumatic conditions in clinical practice and constitute a principal pathological factor contributing to acute pain and motor dysfunction (1, 2). The injury mechanisms exhibit age-specific patterns: high-energy trauma (e.g., traffic accidents, falls from height) predominates in younger populations, whereas osteoporotic low-energy falls are frequently implicated in elderly patients. These fractures commonly involve the distal radius, ankle, and tibia-fibula, typically necessitating surgical internal fixation to restore anatomical alignment and physiological function. Nevertheless, severe postoperative acute pain not only substantially compromises patient rehabilitation but also serves as a critical impediment to quality of life (3).

In the context of multimodal perioperative analgesia, ultrasound-guided peripheral nerve block (PNB) has emerged as a standard component in the management of extremity fracture surgery, attributable to its pronounced advantages of high precision, rapid onset, and minimal local tissue injury. However, with the widespread application of this technique, a characteristic complication—intense pain rebound following PNB pharmacological resolution (4), designated “rebound pain (RP)”—has garnered increasing clinical attention.

Investigations have demonstrated elevated incidence rates of this phenomenon following orthopedic surgical procedures (5–7), with one cross-sectional study reporting RP occurrence in up to 70.9% of patients upon regional anesthesia resolution (8). RP is particularly prevalent following upper extremity brachial plexus blockade or lower extremity femoral and sciatic nerve blockade (9, 10). RP not only exacerbates postoperative opioid consumption but may also precipitate unplanned re-encounters, diminish patient satisfaction, and substantially increase healthcare expenditures.

This review aims to systematically delineate the clinical manifestations, pathophysiological mechanisms, predictive risk factors, and management strategies of RP following extremity fracture surgery. We propose an integrative conceptual framework—the “RP Triple-Hit Model”—to elucidate the interactions among various risk factors, thereby providing theoretical foundations and clinical references for optimizing postoperative pain management and formulating targeted preventive measures.

## 2 Materials and methods

Comprehensive literature searches were conducted in PubMed and Web of Science databases, with retrieval timeframe extending through March 2026. The search strategy employed the following terms: “extremity fracture,” “distal radius fracture,” “ankle fracture,” “tibial fracture,” “femoral fracture,” “humeral fracture,” “peripheral nerve block,” “rebound pain,” “rebound hyperalgesia,” “postoperative pain,” “brachial plexus block,” “interscalene block,” “supraclavicular block,” “infraclavicular block,” “axillary block,” “femoral nerve block,” “sciatic nerve block,” “popliteal block,” “adductor canal block,” “multimodal analgesia,” and “continuous nerve block.”

Inclusion criteria encompassed review articles, clinical trials, randomized controlled trials, prospective and retrospective cohort studies, systematic reviews, and meta-analyses related to PNB, RP, postoperative pain, and analgesia in extremity fracture surgery. Exclusion criteria comprised studies focusing on non-fracture orthopedic procedures, non-regional anesthetic techniques, or investigations lacking specific RP data. Literature screening was performed independently by two investigators, with discrepancies resolved through discussion and consensus.

## 3 Definition, clinical characteristics, and incidence of RP

### 3.1 Definition

**Figure 1:**
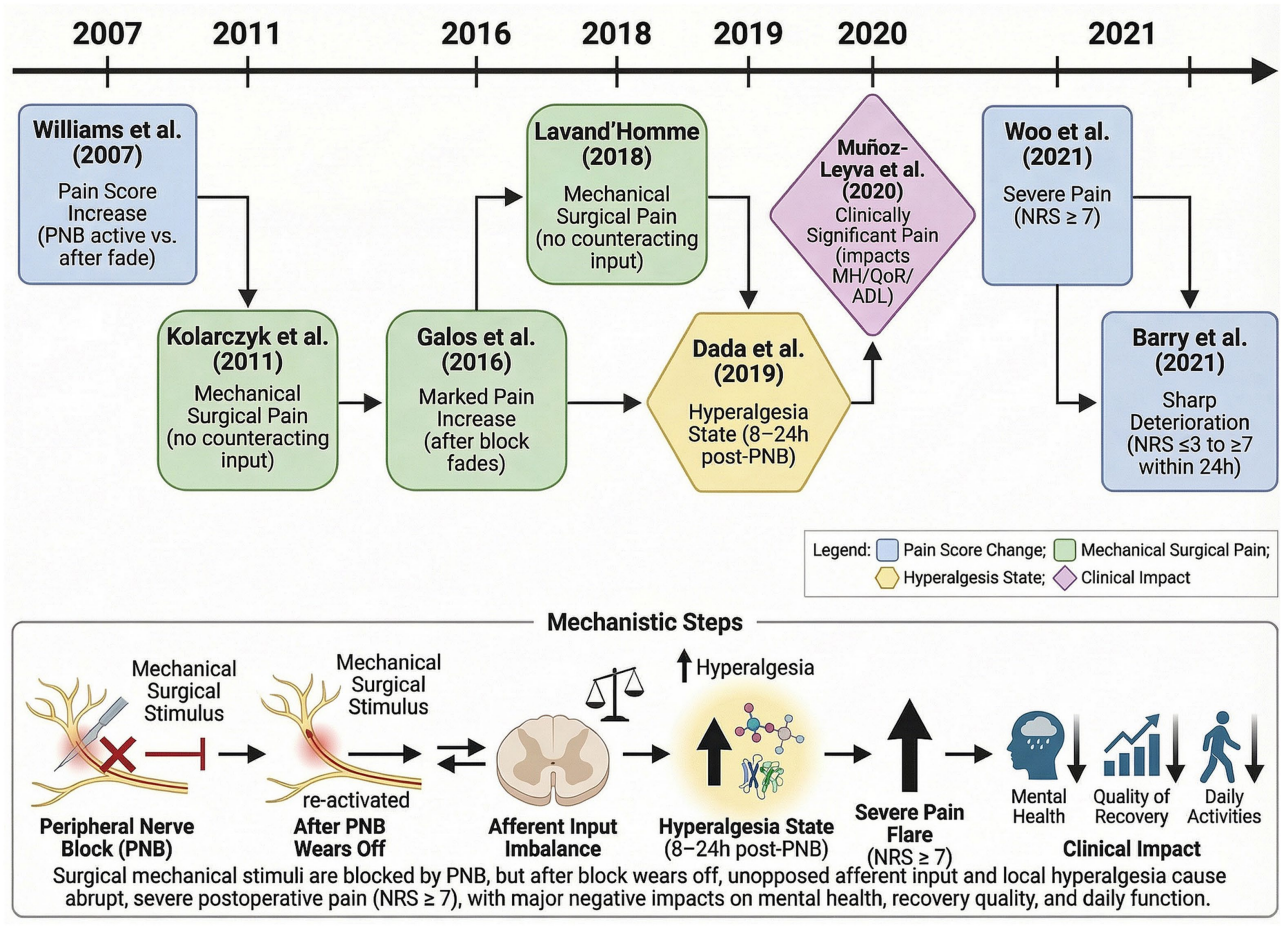
Historical evolution of rebound pain definitions.

Currently, no standardized consensus definition for PNB-related RP has been established in the academic community, with considerable heterogeneity observed across published studies (Figure 1). In 2007, Williams et al. initially introduced this concept (11), defining it as the quantifiable difference in acute pain scores between the effective duration of PNB and several hours following its resolution. Subsequently, Barry et al. (5) proposed a modified scoring methodology with enhanced clinical operability. This approach defines the RP score as the difference between the highest numerical rating scale pain score within 24 h post-PNB and the lowest score recorded in the post-anesthesia care unit. Given its facile implementation and assessment in clinical research, this definition has been adopted and validated in multiple subsequent investigations (12, 13).

### 3.2 Clinical characteristics

The clinical manifestations of RP following extremity fracture surgery exhibit distinctive features, which can be systematically categorized as follows: First, pain is strictly localized to the fracture surgical site and the dermatome distribution of the previously blocked nerve. Second, RP typically emerges approximately 12–24 h following PNB resolution, frequently with acute nocturnal onset, thereby substantially disrupting patient sleep and rest (14). Third, the pain intensity is extraordinarily severe, with patient-reported Numerical Rating Scale (NRS) scores commonly reaching 7–10 (15, 16), corresponding to severe pain ranges. Conventional multimodal analgesic regimens comprising nonsteroidal anti-inflammatory drugs or acetaminophen often prove inadequate, necessitating rescue analgesia with potent opioid agents (17). The pain quality is predominantly characterized by sudden burning sensations or persistent dull aching (18), with individual episodes lasting 2–6 h. Fourth, multidimensional adverse impacts on rehabilitation: severe RP may initiate a vicious cycle between sleep disruption and pain amplification (19). This not only exacerbates psychological distress but also directly compromises postoperative recovery quality and activities of daily living, ultimately resulting in delayed overall rehabilitation progression (20).

### 3.3 Incidence

The incidence of RP following extremity fracture surgery varies significantly according to surgical site and procedure type (5, 6, 21–27) (Table 1).

**Table 1:** Incidence of rebound pain following extremity fracture surgery.

Surgical site	Incidence	Study design	Study period	References
Distal radius fracture (wrist)	12%	Retrospective cohort study	2016	Sunderland et al. (24)
Distal radius fracture	19.00%	Retrospective propensity-matched cohort study	2020–2025	Chao et al. (25)
Ankle/tibial fracture	61.70%	Cross-sectional study	2021–2022	Admassie et al. (23)
Ankle fracture	61.70%	Exploratory pilot study	2019	Sort et al. (26)
Hip/femoral fracture	10% (liposomal bupivacaine group) vs. 43% (control group)	Prospective RCT	2024–2025	Wang et al. (6)
Hip fracture (PENG block)	26.7% (ropivacaine group) vs. 10% (liposomal bupivacaine group)	Prospective RCT	2024–2025	Wang et al. (6)
Upper extremity fracture (brachial plexus block)	40.40%	Prospective RCT	2019–2021	Touil et al. (27)
Upper extremity fracture (brachial plexus block)	49.60%	Retrospective cohort study	2017–2019	Barry et al. (5)
Shoulder surgery (including fractures)	41.2% (control group) vs. 28.6% (dexamethasone group) vs. 23.3% (clonidine group)	Multicenter prospective RCT	2022	Nobre et al. (22)
Wrist fracture surgery	12% (PNB group) vs. 4% (general anesthesia group)	Retrospective quality improvement project	2016	Sunderland et al. (24)

## 4 Underlying mechanisms

RP represents a complex acute pain phenomenon whose underlying mechanisms remain incompletely elucidated. Experimental and clinical investigations suggest that RP may constitute an acute nociceptive sensitization phenomenon arising from spatiotemporally coupled multisignal afferent transmission and multifaceted interactions between peripheral and central neural pathways.

### 4.1 “Unmasking” mechanism (core mechanism)

Although PNB interrupts the conduction of signals from peripheral nociceptors to the spinal cord and supraspinal centers (28), nociceptive stimulation resulting from surgical trauma persists. Upon resolution of the “masking” effect of PNB, accumulated nociceptive responses become “re-exposed,” thereby precipitating intense pain. This “unmasking” mechanism transcends simple hyperalgesia and has been extensively investigated in multiple studies, representing one of the core mechanisms implicated in RP. Following PNB resolution, certain nerve fibers—particularly C-fibers transmitting slow pain—may exhibit aberrant spontaneous hyperexcitability, resulting in pathologically enhanced nociceptive sensitivity and RP manifestation. These characteristics parallel C-fiber hyperactivity observed in neuropathic pain models, subsequently leading to thermal hyperalgesia (29).

### 4.2 Inflammatory response and central sensitization

Fracture itself induces damage to osseous tissue, periosteum, and surrounding soft tissues, resulting in substantial release of inflammatory mediators (30, 31) and algogenic substances; concurrently, surgical incisions trigger acute inflammatory responses (32), augmenting local immune cell infiltration. Multiple inflammatory mediators, including tumor necrosis factor-α, prostaglandin E₂, interleukin-6, and interleukin-1β, become activated, sensitizing peripheral nociceptors (33, 34). Peripheral nociceptive inflammatory signals ascend via nerve terminals to the spinal cord, activating microglia and consequently precipitating release of additional inflammatory molecules, generating cascade reactions that amplify nociceptive signaling (35). Simultaneously, surgical trauma induces sensory remodeling of the nervous system, analogous to a “pain memory” state. Due to persistent peripheral tissue injury-induced inflammatory responses and central sensitization, the remodeled nervous system amplifies afferent nociceptive signals upon withdrawal of PNB protection, thereby producing severe pain (36).

### 4.3 Local anesthetic neurotoxicity

Cellular and animal experimental evidence indicates that local anesthetics possess both neurotoxic (37) and cytotoxic properties. Relevant investigations have demonstrated that local anesthetics can induce neuronal apoptosis in murine models (38). The underlying mechanisms of neural injury are complex, potentially involving cyclooxygenase-2–prostaglandin E₂ pathways, calcium channels, and caspase-mediated pathways (39), thereby exacerbating inflammatory pain and establishing a vicious cycle.

### 4.4 Iatrogenic and patient-related factors

Iatrogenic and patient-related factors warrant consideration. Needle instrumentation trauma, fracture fragment displacement, surgical traction, intraoperative malpositioning, and tourniquet application can all induce neural injury, which represents one potential mechanism underlying RP following PNB (40, 41). Furthermore, patient pain cognition (42), anxiety states (43), and pain catastrophizing have been implicated in amplifying pain experiences upon block resolution.

**Figure 2:**
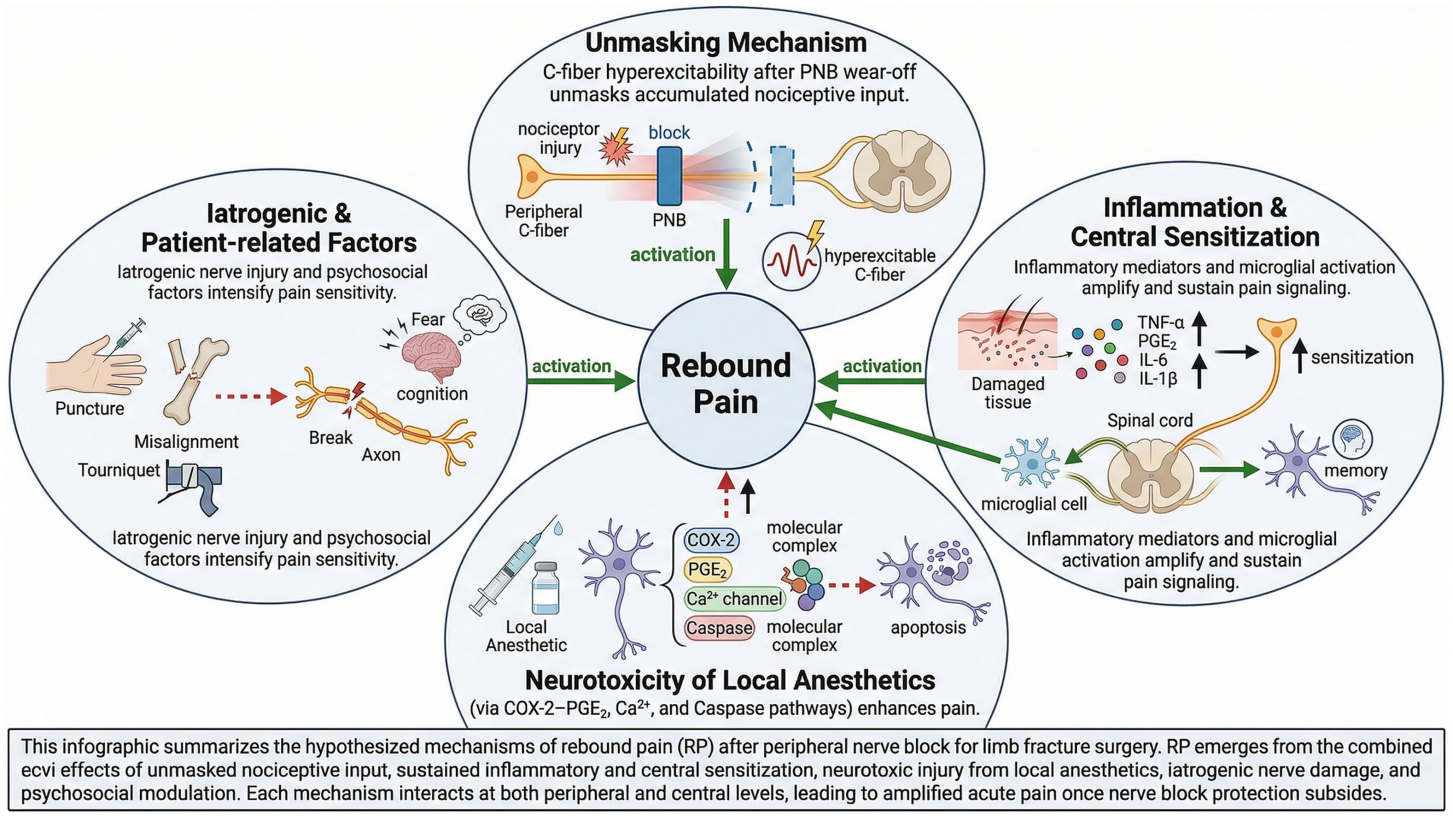
Underlying mechanisms of rebound pain.

Collectively, RP following extremity fracture surgery results from the intricate interplay of multiple mechanisms (Figure 2), encompassing peripheral-central neural interactions, local anesthetic effects, neural plasticity and inflammatory immunity, iatrogenic injury, and psychological-cognitive factors.

## 5 The Triple-Hit Model: an integrative framework for RP risk factors

**Figure 3:**
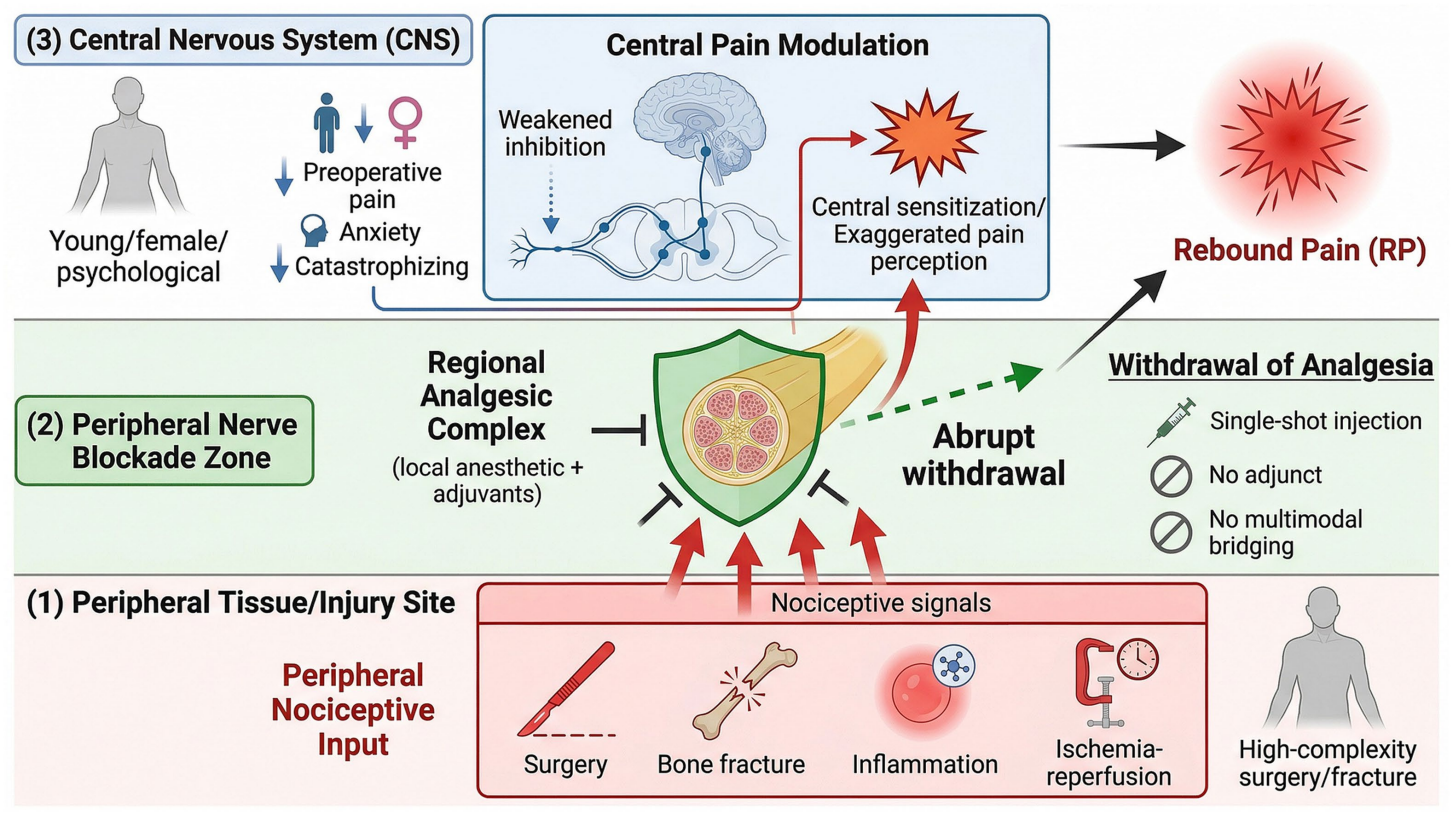
The Triple-Hit Model: a conceptual framework for risk factors of rebound pain.

Understanding the risk factors for RP constitutes the prerequisite for identifying high-risk patients and implementing precision prevention. This review proposes an integrative conceptual framework based on the “RP Triple-Hit Model” (Figure 3), positing that RP emergence results from the synergistic interaction of three convergent factors: the first hit comprises intense nociceptive signal input from peripheral tissues; the second hit involves inadequate processing and modulation capacity of the central nervous system for pain; the third hit encompasses abrupt or inappropriate withdrawal of regional analgesic protection.

### 5.1 First hit: intensity of nociceptive input

Nociceptive input principally includes: First, pain signal load from surgical trauma and anesthetic procedures, correlating with surgical complexity, extent of soft tissue dissection, incision length, and operative duration; more complex and prolonged surgeries induce greater tissue injury and inflammatory responses, thereby elevating RP risk (44). Additionally, regions with dense neural innervation (e.g., brachial plexus, popliteal fossa) permit greater “influx” of nociceptive signals upon block resolution, thereby exacerbating pain rebound (45). Second, more severe osseous and periosteal injury, hematoma formation, and inflammation from the fracture itself result in greater algogenic substance release, conferring substantially higher RP risk compared to isolated soft tissue injury. Third, iatrogenic factors such as tourniquet-induced ischemia–reperfusion injury amplify local inflammation (46); the potential impact of high-concentration, large-volume local anesthetics on neural tissue also correlates with RP, though further investigation is warranted.

### 5.2 Second hit: central nervous system processing capacity

When factors compromising the intrinsic capacity of the central nervous system to process pain signals are present, central sensitization becomes amplified and RP following PNB is more likely to occur (47). Younger patients exhibit lower pain tolerance and diminished capacity to endure pain (48); female sex represents an independent risk factor for RP, potentially attributable to physiological differences, hormonal influences, and psychosocial factors. Preoperative moderate-to-severe pain predisposes the peripheral or central nervous system to a sensitized state (49); contrast effects in pain perception before and after blockade, pain catastrophizing, anxiety states, and patterns of fear and helplessness regarding pain constitute psychological indicators associated with severe RP.

### 5.3 Third hit: mode of analgesic protection withdrawal

The duration of PNB efficacy and the mode of analgesic withdrawal are critical determinants of RP occurrence. First, single-injection block resolution tends to be more abrupt compared to prolonged infusion via continuous catheter, rendering RP more likely and frequently producing a pronounced “pain-free to severe-pain” contrast (50). Second, adjuvant utilization including dexamethasone and ketamine can prolong PNB duration, thereby influencing the temporal profile of analgesia. Finally, effective continuity of adjunctive analgesic medications is essential; timely initiation of multimodal analgesic management prior to block resolution substantially enhances patient pain tolerance thresholds (40).

Based on the Triple-Hit Model, patients at high risk for RP theoretically exhibit the following characteristics: undergoing high-trauma, complex skeletal procedures (strong first hit); young female sex, preoperative pain, or presence of pain catastrophizing (weak second hit/enhanced sensitization); and receipt of single-shot PNB without adjuvants and without timely analgesic bridging protocols (unfavorable third hit modality).

## 6 Preventive strategies for rebound pain following peripheral nerve block in extremity fracture surgery

Based on comprehensive understanding of the pathophysiological mechanisms of RP and the multifactorial risk factors encompassed within the Triple-Hit Model, the formulation of precision, stratified prevention strategies is paramount for optimizing the perioperative experience of patients undergoing extremity fracture surgery.

### 6.1 Optimization and prolongation of peripheral nerve block techniques

Extension of effective analgesic duration represents the cornerstone intervention for RP prevention, principally achieved through continuous peripheral nerve block (CPNB) and combined PNB techniques. CPNB, delivering local anesthetic via indwelling catheter through continuous or patient-controlled infusion, significantly prolongs the analgesic window (51), effectively delaying the emergence and attenuating the peak intensity of pain, thereby substantially reducing the risk of severe early postoperative RP. Clinical investigations have demonstrated that continuous popliteal sciatic nerve block effectively decreases RP and opioid requirements following ankle surgery (52). However, inherent limitations including catheter displacement, infection risk, and management complexity constrain its widespread application in routine clinical practice (53).

Combined PNB strategies, simultaneously blocking multiple innervating nerves or integrating incisional local infiltration, achieve comprehensive analgesic coverage of surgically innervated regions (e.g., shoulder, proximal humerus), thereby effectively circumventing RP attributable to incomplete analgesia (54, 55). The clinical benefits of this approach have been substantiated by randomized controlled trials: in video-assisted thoracoscopic lobectomy, combined erector spinae plane block compared with thoracic paravertebral block alone significantly reduced 24-h postoperative RP incidence (23.64% versus 47.27%), improved activity-related pain, decreased opioid consumption, shortened hospitalization, and enhanced recovery quality (56). Although this evidence derives from thoracic surgery, the core mechanism of multi-target analgesia to eliminate PNB “blind spots” holds significant translational value for fracture surgery, particularly applicable to fracture regions innervated by multiple nerves (e.g., shoulder, proximal humerus, and hip), suggesting that combined blockade strategies may represent an effective approach for reducing RP risk following extremity fracture surgery.

### 6.2 Application of adjuvants

Adjuvant utilization aims to enhance local anesthetic efficacy, prolong duration of action, or mitigate neurotoxicity through pharmacological mechanisms. Dexamethasone, the most extensively investigated adjuvant (57, 58), may exert its effects through local anti-inflammatory actions, suppression of nociceptive C-fiber excitability, and antagonism of potential local anesthetic-induced neurotoxicity (59). For RP prevention, both intravenous and perineural administration significantly reduce RP incidence (RR = 0.38, 95%CI 0.28–0.51) (60), with intravenous route demonstrating superior evidence quality (SUCRA = 0.95) (61). Specifically, intravenous dexamethasone 5–8 mg (62) reduces RP incidence from 36 to 22% following arthroscopic shoulder surgery (18), while combined adductor canal block and IPACK block (4 mg each) in total knee arthroplasty decreases RP incidence from 30 to 5% (63). Although perineural administration (64) (e.g., direct admixture with ropivacaine for supraclavicular brachial plexus block) prolongs block duration by approximately 3–4 h compared with intravenous route (22.81 h versus 18.92 h) (7), combined administration of both routes fails to demonstrate additive or synergistic effects (58), with no significant differences in 24-h postoperative pain scores or opioid consumption (7). Notably, in patients receiving robust multimodal analgesia (NSAIDs plus acetaminophen), the incremental benefit of dexamethasone may be obscured by a “ceiling effect” (65). The recommended dosage is 4–10 mg; in resource-limited settings, it represents the most effective independent protective factor against severe RP (AOR = 0.24) (8), with favorable safety profile, minimal impact on glycemic control, and no increased infection risk. Despite recent challenges, current consensus supports its beneficial role in RP prevention (60), though optimal route and dosage remain active research foci (61).

Dexmedetomidine, an α₂-adrenergic receptor agonist, possesses sedative, anxiolytic, and synergistic analgesic properties, prolonging analgesic duration and attenuating inflammatory responses, though vigilance for bradycardia and oversedation is warranted (66). Ketamine, an N-methyl-D-aspartate receptor antagonist, may exert effects through central sensitization inhibition: perineural esketamine prolongs block duration and reduces RP incidence (67); intraoperative intravenous administration, while failing to significantly reduce RP incidence following arthroscopic shoulder surgery (~25%), improves resting pain scores at 8–24 h and enhances hemodynamic stability (68); meta-analysis demonstrates that intravenous or perineural ketamine/esketamine significantly reduces PNB-related RP incidence (OR 0.48, 95% CI 0.38–0.60), improves postoperative 12–48 h pain scores, without increased adverse events, though limited by study quantity and clinical heterogeneity, necessitating further high-quality trials (69). Buprenorphine, a partial *μ*-opioid receptor agonist, demonstrates potential for prolonged analgesia and RP reduction at high perineural doses (>300 μg) (70). Liposomal bupivacaine, despite providing analgesia lasting several days, currently lacks evidence supporting superiority over conventional local anesthetics for RP prevention. Animal experiments indicate that while it prolongs analgesic duration, it fails to prevent RP, merely delaying its onset, and is associated with more severe Wallerian degeneration and perineural inflammation (71).

Notably, the aforementioned evidence for adjuvant agents is predominantly derived from non-fracture surgical populations (e.g., arthroscopic shoulder surgery, knee arthroplasty), and direct extrapolation to fracture patients remains uncertain. Fracture patients exhibit distinct perioperative characteristics, including acute inflammatory responses, preoperative pain sensitization, and psychological stress, which may substantially differ from elective surgical cohorts and potentially modulate adjuvant efficacy. Although the core pharmacological mechanisms of these agents—enhancement of local anesthetic potency, prolongation of neural blockade duration, and modulation of central sensitization—hold theoretical relevance for fracture surgery, optimal dosing regimens and magnitude of effect require validation in fracture-specific studies. At present, cautious application of adjuvants in extremity fracture surgery is recommended, with vigilant monitoring for adverse effects.

### 6.3 Multimodal analgesic strategies

Multimodal analgesia constitutes the fundamental principle for RP prevention, grounded in the theoretical foundation that combined application of analgesic agents and techniques with divergent mechanisms of action before and after PNB resolution achieves multi-target synergistic blockade of pain signal transduction pathways, thereby producing additive or synergistic effects.

A prototypical multimodal regimen typically comprises the following components: continuous PNB or combined/compound PNB techniques, prolonging the analgesic window or achieving comprehensive surgical site neural coverage, effectively delaying pain peak emergence and attenuating peak intensity (72); nonsteroidal anti-inflammatory drugs, inhibiting surgery-induced local inflammatory cascade reactions and reducing algogenic substance production at the source; acetaminophen, as a foundational analgesic, providing basal analgesic effects through central and peripheral mechanisms; calcium channel modulators such as gabapentin or pregabalin, regulating voltage-gated calcium channels and inhibiting central sensitization processes, considered integral components of “preventive analgesia,” though potential adverse effects including sedation and dizziness require careful consideration (73). Additionally, as “rescue analgesia” measures, short-acting opioids are routinely prepared for emergent severe pain.

Precise timing of drug administration is critical for ensuring multimodal analgesic efficacy. All oral analgesic medications should be administered 1–2 h before anticipated PNB resolution, ensuring that when nociceptive stimulation re-emerges, systemic analgesic drug concentrations have achieved therapeutically effective levels, thereby realizing seamless analgesic continuity and avoiding severe “unmasking” pain attributable to analgesic gaps (51).

### 6.4 Patient education

Patient education represents the most cost-effective and modifiable intervention within the preventive system (14). Comprehensive preoperative education significantly modulates patient pain perception patterns and stress responses.

Educational content should systematically encompass the following dimensions: explicit explanation of the time-limited nature of PNB analgesia with estimated resolution time; objective description of RP timing, characteristics, and intensity, establishing realistic psychological expectations and reducing anxiety stemming from fear of the unknown; emphasis on the paramount importance of timely administration of preventive analgesic medications before block resolution; detailed demonstration of correct usage for all prescribed medications; and provision of clear healthcare contact pathways ensuring professional assistance when analgesic efficacy is inadequate (74).

Through multi-dimensional, repetitive education combining verbal instruction, written materials, and multimedia tools, not only can perioperative anxiety be effectively reduced, but compliance with analgesic regimens can be significantly enhanced, thereby indirectly reducing the risk of severe RP (75).

## 7 Conclusion

PNB represents an important adjunct for anesthesia and analgesia in extremity fracture surgery; however, a subset of patients experiences severe pain following block resolution, a clinical phenomenon frequently underestimated in practice. Based on available evidence, this review proposes the “Triple-Hit Model” of RP following PNB in extremity fracture surgery, conceptualizing RP as a dynamic outcome of the interaction among peripheral nociceptive input intensity, central nervous system processing capacity, and characteristics of analgesic protection withdrawal.

This integrative framework provides the following clinical applications: first, risk stratification, identifying high-risk patients (e.g., young females, significant preoperative pain, complex procedures, single-shot high-volume blockade without adjuvants); second, stratified intervention, guiding targeted preventive strategies including reducing peripheral nociceptive input through anti-inflammatory approaches, enhancing central processing capacity through psychological support and preventive analgesia, and optimizing analgesic protection withdrawal through continuous or combined blockade techniques.

Key clinical recommendations include: routine implementation of multimodal analgesic protocols initiated before block resolution; prioritization of continuous catheter techniques or combined blockade for high-risk patients; rational application of dexamethasone as a local anesthetic adjuvant; enhanced patient education regarding expected pain trajectory and medication timing.

As a narrative review, this work is limited by lack of systematic literature search and quantitative data integration. The relative weights of individual components may vary according to interindividual differences and clinical scenarios, and their interactions may exhibit nonlinear characteristics and threshold effects. Future research should focus on prospective cohort validation of this model and exploration of more precise predictive indicators.
